# Lingual Leiomyosarcoma: A Histopathological Case Report

**DOI:** 10.7759/cureus.32717

**Published:** 2022-12-20

**Authors:** Hristo Popov, Lilyana Petkova, George S Stoyanov

**Affiliations:** 1 General and Clinical Pathology/Forensic Medicine and Deontology, Medical University of Varna, Varna, BGR; 2 General and Clinical Pathology, St. Marina University Hospital, Varna, BGR

**Keywords:** pathology, leiomyosarcoma, tongue, spindle shape tumor, head and neck malignancy

## Abstract

Leiomyosarcomas are rare malignant mesenchymal neoplasms originating from smooth muscle cells. Although leiomyosarcoma is commonly located in the female reproductive system, gastrointestinal tract, and subcutaneous tissues, it is a rare entry in the head and neck area, probably due to the scarcity of smooth muscle tissue in this topographical region. Herein we present a histopathological case report of a 60-year-old male with a slow-growing painless mass on the middle third of the right lateral lingual margin, with focal ulceration measuring 1x1.5cm. After gross excision, histopathology revealed pleomorphic spindle cells, some with bizarre nuclei and abundant pathological mitotic activity with a tendency to grow in a fascicular pattern. As the patient had the demographic characteristic and risk factors for oral cavity squamous cell carcinoma, a spindle-shaped variety (sarcomatoid) variety was suspected, and immunohistochemistry with a broad set of antibodies was used to prove the histogenetic group of the tumor. As the tumor was pan-cytokeratin and desmin negative, focally positive for caldesmon, and positive for smooth muscle actin, the diagnosis of pleomorphic leiomyosarcoma of the tongue was established.

## Introduction

Malignant mesenchymal tumors represent around 0.7% of all malignant neoplasms, and leiomyosarcomas account for only 5% to 10% of those [1,2]. The most common sites for leiomyosarcoma development are the myometrium, gastrointestinal tract, and skin [3].

The highest incidence rates are in the age group between 40 and 49 years. While most common in this age group, leiomyosarcoma can also develop in the pediatric population where the prognosis is significantly more favorable. Women are more commonly affected than men due to the significant incidence of this nosological unit within the uterus [4].

Leiomyosarcomas are exceedingly rare in the head and neck area, especially the oral cavity and oropharynx, but when they develop in these locations, the tongue is the most commonly affected location, followed by the lips and palate [5]. The prognosis is generally poor with a high recurrence and/or metastasis rate. The most common sites of metastasis are the lungs, bones, and brain [6]. As with most mesenchymal tumors, lymph node metastasis is exceedingly rare [6].

Presenting symptoms of leiomyosarcoma within the oral cavity are typically those of a painless, slow-growing tumor formation [7]. The main late symptoms specific to the part of the oral cavity affected are pain, loose teeth, and difficulty chewing and talking [8]. Discoloration and ulcers of the tongue can also develop in the latter stages of tumor evolution. Epistaxis, dysphagia, hoarseness, fever, stridor, and cough have also been reported as late complications associated with local invasion and are again dependent on the specific site or origin i.e., the palate, the base of the tongue, and gingival margins [2,7-9]. Due to the nonspecific clinical appearance, the diagnosis is made after histological examination with more common malignancies often being the suspected clinical culprit, such as squamous cell carcinoma.

Histomorphologically, the tumor is comprised of atypical spindle cells with characteristic nuclei with truncated ends growing in intertwining bundles. Nuclei may be palisading, and often, myofibrils are recognizable in more differentiated tumors [4]. Anaplastic features such as large and monstrous cells, pyknotic nuclei, mitotic figures, and necrosis are variably present. Furthermore, other than the conventional forms some rare forms of leiomyosarcoma have also been identified based on their morphology, such as giant cell leiomyosarcoma, pleomorphic leiomyosarcoma [4], inflammatory leiomyosarcoma [10], epithelioid leiomyosarcoma, and desmoplastic leiomyosarcoma.

Immunohistochemical verification is essential for defining the diagnosis based on these varying histomorphological subtypes, variants, and degrees of differentiation. Due to the vast set of differentials, based on the histological appearance of the tumor alone, the use of a broad panel of antibodies, including actins (smooth muscle: SMA and HHF-35), desmin, vimentin, cytokeratins, and S-100 protein, should be undertaken to differentiate from sarcomatoid squamous cell malignancies, rhabdomyosarcoma, fibrosarcoma, variants of liposarcoma and other rare mesenchymal tumors [9].

After diagnosis, the recommended treatment for leiomyosarcoma is primarily surgical, consisting of wide local excision and adjuvant therapy (radiotherapy), with some studies reporting no added survival benefit with the addition of chemotherapy [11].

## Case presentation

A 60-year-old male presented to our healthcare institution with a palpable, semi-exophytic mass located on the middle third of the right lateral lingual margin with focal ulceration. The finding persisted for several months with steady growth; by the time of presentation, the gross tumor size was 1x1.5cm.

Previous medical history included hypertensive disease for the past 15 years under adequate medication control but was otherwise uneventful. Risk factors for head and neck neoplasia included tobacco smoking for the past 20 years (25 pack-years). As the lesion was suspicious for oral cavity squamous cell carcinoma, the patient was scheduled for surgery with wide gross excision of the tumor mass. The specimen sent for histopathology showed an ulcerated invasive mass, grossly non-involving the lingual resection margins.

**Figure 1:**
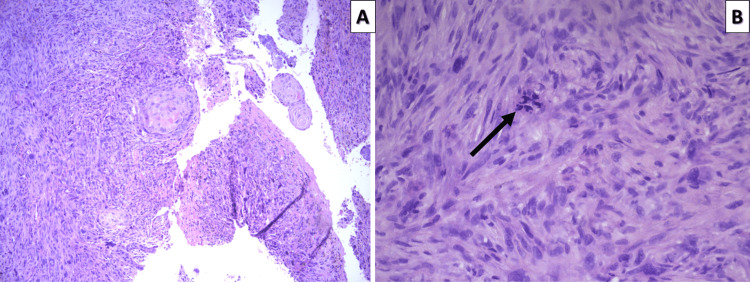
Histomorphology of the tumor specimen A: Fascicular growth pattern and focal squamous cell remnants from the oral cavity mucosa; hematoxylin and eosin stain, original magnification 100x B: Spindle cells, some with truncated nuclear ends, growing in an intertwining fascicular pattern and a pathological mitotic figure (arrow); hematoxylin and eosin stain, original magnification 400x

Morphology of the tumor revealed a spindle cell neoplasm with high mitotic activity, bizarre and monstrous cells, with predominantly intersecting fascicular morphology (Figure 1).

**Figure 2:**
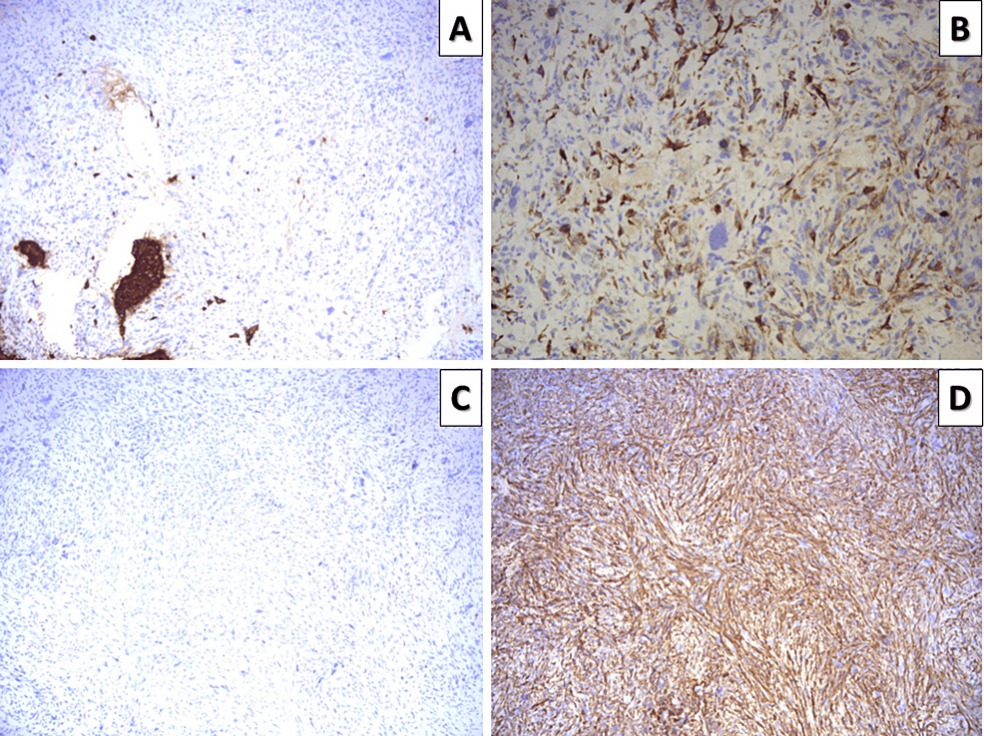
Immunophenotype of the tumor A: Pan-cytokeratin, no expression from the tumor cells. Note that focal expression is due to mucosal remnants in the specimen, original magnification 100x; B: Focal Caldesmon expression, original magnification 200x; C: No expression of desmin from the tumor cells, original magnification 100x; D: Smooth muscle actin is diffusely positive in the tumor cells, original magnification 100x

As the patient had risk factors and the tumor morphology was non-conventional with some remnants of squamous epithelia present superficially with dysplastic changes, the diagnosis of spindle shape squamous cell carcinoma was suspected; however, a broad set of immunohistochemical markers were included as differentials. The tumor was negative for pan-cytokeratin (CK AE1/AE3), disproving its epithelial origin. Focal expression for caldesmon was noted, with desmin negativity and a diffuse strong cytoplasmic expression of smooth muscle actin (SMA), confirming the diagnosis of leiomyosarcoma of the tongue (Figure 2). The histological features showed significant polymorphism, so the tumor was classified as a pleomorphic leiomyosarcoma. The Ki-67 proliferative index was 60%. The resection margins were non-involved, and the tumor was staged as pT2.

After the diagnosis was established, the patient underwent positron emission tomography, which showed ipsilateral lymphadenopathy with involvement of a single submandibular lymph node and borderline activity in the IIA level region, suggestive of metastatic dissemination. Although stable on follow-up and undergoing chemo and radiotherapy, the patient was lost to follow-up six months after surgery.

## Discussion

Leiomyosarcoma is a malignant tumor that originates from smooth muscle cells and most often occurs in the uterus, gastrointestinal tract, retroperitoneum, skin, and subcutaneous tissues. It rarely develops in the head and neck region, probably due to the small amount of smooth muscle tissue in this region [6].

The most common sites of origin of head and neck leiomyosarcoma are the blood vessels, ductus lingualis, circumvallate papillae, myoepithelial cells, or undifferentiated mesenchymal cells situated in the deep tissues [2,7-9,11]. Primary leiomyosarcoma of the tongue is a rare tumor entry [3]. To date, there are around 20 reported cases in the medical literature of primary leiomyosarcoma of the tongue [3,6,8,9]. The cause of leiomyosarcoma in this location remains widely unknown, although cases have been linked to trauma, smoking, estrogen stimulation, ionizing radiation, and the Epstein-Barr virus [12,13].

As underlined by our case, the histopathological diagnosis after gross excision is impossible based on morphology alone. Initially, the atypical spindle cells, atypical mitoses, and remnants of squamous mucosa led us to suspect sarcomatoid (spindle shape) squamous cell carcinoma, as the patient was in an age group and had risk factors suggestive of this malignancy. Immunohistochemistry, however, disproved the epithelial nature of the neoplasm and showed the presence of smooth muscle filaments, while desmin negativity disproved rhabdomyosarcoma and some other mesenchymal tumors.

The diagnosis of leiomyosarcoma in this topographical location, as in most other locations, is based not only on histopathological but predominantly on immunohistochemical criteria [4,10]. Histologic criteria are represented by a structural pattern of intertwining bundles of atypical cells, some with smooth muscle morphology, high mitotic activity, pleomorphism, and atypical cell shapes indicative of a malignant spindle cell tumor not only from the mesenchymal histogenic group [4]. The differential diagnosis includes several types of fusiform tumors and can be extremely difficult when these tumors show high-grade anaplasia and/or unusual localization, such as squamous cell carcinoma, melanoma, other mesenchymal tumors, and, in some cases, vascular tumors. As such, the immunohistochemical pattern of expression of the aforementioned specific antibodies and lack thereof of antibodies specific for other entries is the primary method to confirm the diagnosis, although the genetic profile of these tumors is also somewhat specific [4,10,14].

As already mentioned, sarcomas or malignant soft tissue tumors are rare neoplastic entries, especially in the head and neck area [6]. Based on their histological profile, they broadly comprise the majority of spindle cell tumors and require a broad set of differentials. The fifth edition of the World Health Organization's classification of head and neck tumors lists adipocytic, fibroblastic, skeletal and smooth muscle, vascular and perycytic, peripheral nerve, chondro-osseus, undifferentiated and tumors with an uncertain differentiation in the sarcoma family [15]. The malignant entities from the specified groups present a varying degree of spindle cells on histopathology and require a broad differential. Furthermore, other soft and non-soft tissue tumors such as solitary fibrous tumors, spindle cell achromatic melanoma, the aforementioned spindle cell variety of squamous cell carcinoma, and metastatic lesions although rare to the oral cavity, should also be included in the histopathological differential diagnosis [15,16]. In our case, the initial set of antibodies proved sufficient in their expression pattern to define the proper diagnosis while if negative, they would have narrowed down the diagnostic groups with further immunohistochemistry to prove the nosological unit [15].

Furthermore, although conventional histopathology alone is limited in this diagnostic area, it does underline conventional morphological stigmas such as staghorn blood vessels, predominantly for solitary fibrous tumors, rhabdoid differentiation for rhabdomyosarcoma, lypoblasts for liposarcoma, and small blood vessel formation for angiosarcomas [15]. The lack of these features alone indicated undifferentiated or poorly differentiated entries or fibro and leiomyosarcoma predominantly [15].

## Conclusions

Histological verification of neoplasms is an essential part of modern medicine. As seen in our case, even though the patient had classical risk factors, fit the age group and the clinical and gross characteristics of squamous cell carcinoma, and although conventional histopathology could also be interpreted as suggestive of an epithelial malignancy, immunohistochemistry showed origin from a different histogenic group. Although, as reported in the literature, there is a significant rarity of leiomyosarcoma in the oral cavity, it is highly likely that in the absence of immunohistochemistry, a significant amount of these tumors are interpreted as spindle cell squamous cell carcinoma. Hence the actual frequency of these tumors could be significantly higher, and it would be wise to suspect a non-epithelial malignancy in the case of spindle-shaped oral cavity tumors, even in the presence of focal conventional squamous cell morphology.
